# Assessing Anti-Adipogenic Effects of Mango Leaf Tea and Mangiferin within Cultured Adipocytes

**DOI:** 10.3390/diseases11020070

**Published:** 2023-05-10

**Authors:** Sepideh Alasvand Zarasvand, Vivian Haley-Zitlin, Olanrewaju Oladosu, Ikechukwu Esobi, Rhonda Reigers Powell, Terri Bruce, Alexis Stamatikos

**Affiliations:** 1 Department of Food, Nutrition and Packaging Sciences, Clemson University, Clemson, SC 29634, USA; salasva@clemson.edu (S.A.Z.); vivianh@clemson.edu (V.H.-Z.); oolados@g.clemson.edu (O.O.); iesobi@g.clemson.edu (I.E.); 2 Clemson Light Imaging Facility, Clemson University, Clemson, SC 29634, USA; rhondar@clemson.edu (R.R.P.); terri@clemson.edu (T.B.)

**Keywords:** adipogenesis, de novo lipogenesis, diabetes, insulin, in vitro, polyphenol, xanthone

## Abstract

Obesity is a condition caused by surplus adipose tissue and is a risk factor for several diet-related diseases. Obesity is a global epidemic that has also been challenging to treat effectively. However, one promoted therapy to safely treat obesity is anti-adipogenic therapeutics. Therefore, identifying potent anti-adipogenic bioactive compounds that can safely be used clinically may effectively treat obesity in humans. Mango leaf has potential medicinal properties due to its many bioactive compounds that may enhance human health. Mangiferin (MGF) is a primary constituent in mango plants, with many health-promoting qualities. Therefore, this study investigated the effect of MGF, and tea brewed with mango leaves in cultured adipocytes. The anti-adipogenic efficacy of mango leaf tea (MLT) and MGF in 3T3-L1 cells were assessed, along with cell viability, triglyceride levels, adiponectin secretion, and glucose uptake analyzed. In addition, changes in the mRNA expression of genes involved in lipid metabolism within 3T3-L1 cells were determined using quantitative real-time PCR. Our results showed while both MLT and MGF increased glucose uptake in adipocytes, only MLT appeared to inhibit adipogenesis, as determined by decreased triglyceride accumulation. We also observed increased secretory adiponectin levels, reduced ACC mRNA expression, and increased FOXO1 and ATGL gene expression in 3T3-L1 cells treated with MLT but not MGF. Together, these results suggest that MLT may exhibit anti-adipogenic properties independent of MGF content.

## 1. Introduction

Adipogenesis is the process of adipocyte hypertrophy and/or hyperplasia within adipose tissue and can be initiated by excessive energy consumption and/or physical inactivity. Chronic, unregulated adipogenesis drastically increases the risk of obesity in humans and is considered a pandemic in Western societies [1,2]. Obesity also increases the risk of developing both type 2 diabetes mellitus and cardiovascular disease, which are considered major public health issues within industrialized nations [3]. 

Regarding obesity, this condition is considered to be a significant factor in mortality and morbidity/disability, and obesity rates are predicted to increase in number globally. Indeed, obesity is now considered a more significant global health concern than famine [4]. However, obesity is mainly regarded as preventable and treatable in most patients afflicted with this condition, with changes in diet and exercise intervention being effective strategies for preventing and treating obesity [5].

Other options for treating obesity include drug therapy, including the medications orlistat, phentermine, and sibutramine. However, due to the severe side effects of these drugs [6,7,8], researchers have investigated natural products with anti-obesity/anti-diabetic actions that are considered safer options [9], including polyphenolic compounds. Polyphenolic compounds are found widely in plants. Polyphenolic compounds are reported to have anti-obesity activities that may derive from their effect on activation or inactivation of gene expression of transcription factors involved in lipid metabolism that include peroxisome proliferator-activated receptor γ (PPARγ), CCAAT-enhancer-binding proteins (C/EBPα), fatty acid synthase (FAS), adipose triglyceride lipase (ATGL), and forkhead transcription factor class O1 (FOXO1), Acetyl CoA carboxylase (ACC) [10]. 

Adipocytes modulate adipose mass and can provide insight into how adipose tissue responds to lipogenic and lipolytic compounds but are prone to being challenging to culture ex vivo. However, the cell line 3T3-L1 cells are considered a well-established in vitro model for assessing adipocyte biology and physiology. Indeed, a plethora of research has been conducted by utilizing 3T3-L1 cells as an in vitro model to assess bioactive compounds that may alleviate obesity and complications related to obesity, including insulin resistance, glucose intolerance, and systemic inflammation [11,12,13]. 

For example, several studies have investigated anti-adipogenic activities of polyphenolic compounds when using 3T3-L1 cells via measuring the expression of several genes associated with glucose/lipid metabolism, including FAS, ATGL, FOXO1, and ACC, when this cell line is exposed to these types of compounds. For example, when 3T3-L1 cells were treated with the carotenoid lutein, lipid accumulation appeared suppressed via increased FOXO1, ATGL, and HSL compounded with decreased expression of SREBP-1, FAS, and ACC [14]. 

Natural bioactive compounds are historically a prominent source for nutraceutical and pharmaceutical companies. Mango leaf from *Mangifera indica* L. is a natural product containing several polyphenols, including mangiferin (MGF) [15]. Several pharmacological effects of the whole mango plant include antioxidant, anti-inflammatory, analgesic, and anti-adipogenic properties [16,17,18]. These health-promoting properties possibly arise from the high content of MGF and several other polyphenols and bioactive compounds present, including but not limited to methyl gallate, quercetin, catechins, ellagic acids, gallic acid, anthocyanins, carotenoids, and ascorbic acid [15]. While these beneficial compounds are present in mango plants, it has been postulated that the main bioactive ingredient in the mango plant, especially the mango leaves, is MGF [19,20]. Indeed, MGF is touted to demonstrate anti-viral, anti-oncogenic, anti-diabetic, anti-obesity, and hypo-lipidemic properties [21]. However, research is scant on directly assessing the potential anti-adipogenic impact of mango leaf tea (MLT) versus MGF alone. Therefore, this study aimed to evaluate the anti-adipogenic properties of MLT and MGF alone in 3T3-L1 adipocytes. 

## 2. Materials and Methods

### 2.1. Sample Preparation

The mango leaves were washed, dried, crushed, and tea was prepared based on the adapted method from a previous study [22]. Tea was filtered with Whatman disposable sterile syringe filter, pore size: 0.2 µm (GE Healthcare UK Limited, Buckinghamshire, UK), and stored at −20 °C until used. 

### 2.2. Mangiferin Quantification

**Figure 1:**
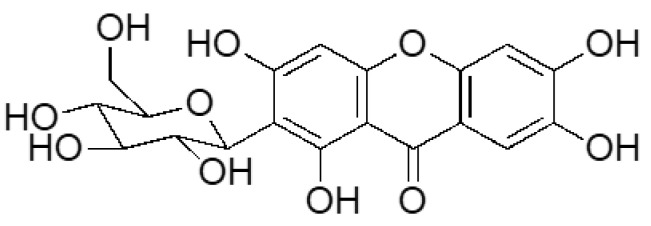
Chemical structure of mangiferin (MGF).

The mangiferin (Figure 1) concentration of representative samples of tea was determined using High-Performance Liquid Chromatography (HPLC) [22], giving an estimated mangiferin concentration similar to previous reports [15]. For treatments, cells were exposed to a ≥98% purified dose of MGF (Sigma-Aldrich. St. Louis, MO, USA) and an approximate concentration of MGF derived from MLT, as previously described [23].

### 2.3. Cell Culture and Adipocyte Differentiation

Mouse 3T3-L1 pre-adipocytes were purchased from American Type Culture Collection (Manassas, VA, USA). Cells were maintained in standard growth medium consisting of high-glucose Dulbecco’s Modified Eagle’s Medium (DMEM; Corning, New York, NY, USA) supplemented with FB Essence (10%; VWR Life Science Seradigm, Radnor, PA, USA) and penicillin–streptomycin (P/S; 1%; Corning) at 37 °C with 5% CO_2_ in 10 cm tissue culture dishes. The 3T3-L1 differentiation protocol was adapted [24] by replacing FBS with FB Essence. In brief, sub-cultures of 3T3-L1 cells were grown in basal media, which contained DMEM supplemented with FB Essence (10%) and P/S (1%), for three days. Next, we washed the cells with PBS. Then we refed cells with differentiation media I contain DMEM supplemented with FB Essence (10%), P/S (1%), 0.5 mM IBMX (3-isobutyl-1-methylxanthine) (MP Biomedicals, OH, USA), 0.25 µM dexamethasone, and 1 µg/mL insulin. After 48 h (day 5), cells were washed with PBS and refed with differentiation media II containing DMEM supplemented with FB Essence (10%), P/S (1%), and 1 µg/mL insulin. After 48 h (day 7), cells were rewashed with PBS and replenished with basal media. For interventions, treatments with vehicle only, MGF (80 µL/10 mL media), and MLT (500 µL/10 mL) were added to differentiation media I and II on days 3 and 5.

### 2.4. MTT Assay

We used an MTT assay kit (BioVision, Milpitas, CA, USA) to evaluate cytotoxicity in undifferentiated 3T3-L1 cells treated with either MLT or MGF. The MTT assay was carried out: PBS-washed cells were initially trypsinized, transferred to 96-well treatment dishes, and cultured in a standard growth medium. Once cells reached 70–80% confluency in the treatment dishes, we washed cells with PBS, then treated cells for 48 h with either vehicle only or increasing concentrations of either MGF or (25, 50, 75, 100, and 125 mM), diluted in standard growth medium. After respective treatments, we washed cells with PBS and incubated the cells with MTT reagent diluted and in serum-free medium for 3 h. We subsequently added MTT solvent to the cells, wrapped the treatment plates with aluminum foil, and exposed the plate to gentle shaking for 15 min. Formazan detection was analyzed by reading absorbance at 590 nm using a SpectraMax^®^ M2 multi-detection microplate reader (Molecular Devices, San Jose, CA, USA) [25].

### 2.5. Measurement of Cellular Triglyceride Content

On day 7 of differentiation, cells were washed with PBS, trypsinized and transferred into 96-well plates with a standard growth medium. After 24 h incubation, we rinsed cells with PBS, then added RIPA lysis buffer (VWR Life Science) to cells to initiate cell lysis. Samples were heated for 5 min at 85 °C and allowed to cool down at room temperature; this step was repeated twice to encourage triglyceride solubilization. Debris was pelleted by centrifugation at 14,000× *g* for 2 min. The supernatants were then assayed for triglyceride content using a Triglyceride Quantification Colorimetric assay kit (Biovision, Milpitas, CA, USA). Colorimetric measurements were performed at 590 nm using an ELISA microplate reader.

### 2.6. Oil-Red O Staining

On day 7 of differentiation, cells were washed twice in PBS and then fixed in 4% paraformaldehyde/PBS for 10 min at room temperature. Cells were then stained with Oil Red O (ORO, Sigma-Aldrich, St. Louis, MO, USA) per the manufacturer’s suggested protocol. Briefly, cells were washed in PBS and then incubated in 60% isopropanol for 5 min. Samples were then stained for 20 min with a working solution of ORO. Cells were washed three times in water and then counterstained with DAPI. Cells were washed twice in water and mounted in PBS: Glycerol (50%/50% *v*/*v*). Samples were imaged using a Leica SPE confocal microscope (Leica Microsystems, Buffalo Grove, IL, USA) using a 63× objective (N.A. = 1.3) and 1.5× zoom. We used a 532 nm excitation laser to visualize ORO and collected emission wavelengths from 555 to 770 nm. To visualize DAPI, we used a 405 nm excitation wavelength and collected emission wavelengths from 415 to 480 nm. Images were gathered using Leica LAS X (version 3.5.2.18963 software).

### 2.7. qRT-PCR

On day 7 of differentiation, we rinsed cells with PBS, lysed cells with TRI Reagent (Zymo Research, Irvine, CA, USA), and then purified total RNA using a Direct-zol RNA purification kit (Zymo Research, Irvine, CA, USA). The total RNA was quantified using a SpectraMax^®^ QuickDrop™ Micro-Volume Spectrophotometer (Molecular Devices, LLC., SanJose, CA, USA) and 500 ng of total RNA to convert mRNA into cDNA by using a qScript cDNA SuperMix kit (Quantabio, Beverly, MA, USA). We used this synthesized cDNA as a template for qPCR, with a Quantabio PerfeCTa SYBR Green FastMix kit being utilized for our qPCR reactions. The primer pairs for target genes and GAPDH reference gene are shown in Table 1. We performed our qPCR reactions using a qTOWER^3^ G touch qPCR instrument (Analytik Jena US), and the qPCR data were analyzed using the ΔΔ^CT^ method [26,27,28].

### 2.8. Measurement of Secretory Adiponectin Levels

On day 7 of differentiation, cell culture media from each treatment was collected and then centrifuged at 1000× *g* for 5 min to pellet any cellular debris. We measured adiponectin in the centrifuged cell culture medium using a mouse adiponectin ELISA kit (AccuSignal^TM^ ELISA Kit, KOA0366; Rockland, Limerick, PA, USA), following the manufacturer’s instructions. 

### 2.9. Cellular Glucose Uptake

We assessed cellular glucose uptake using a Cayman Chemical Glucose Uptake Cell-Based Assay Kit following the manufacturer’s protocol (Ann Arbor, MI, USA). Fluorescently labeled deoxyglucose analog 2-NBDG was the labeled glucose utilized for gauging cellular glucose uptake, and fluorescence was analyzed with a microplate reader. In brief, on day 7 of differentiation, cells were washed with PBS, trypsinized, and then transferred to a 96-well black, transparent bottom culture plate. After 24 h, the medium was discarded, and cells were washed with PBS, followed by incubating cells with a mixture of KRPS and 2-NBDG in a 100:1 ratio, and cells were incubated at 37 °C for 2 h. The plate was then centrifuged for 5 min at 400× *g* at room temperature, and the supernatant was discarded. We then washed cells with PBS and then incubated the cells with Cell-based Assay Buffer before measuring fluorescence under excitation/emission of 485/535 nm. 

### 2.10. Statistical Analysis

We used SigmaPlot (Systat Software Inc, San Jose, CA, USA) to analyze statistics. We conducted a Shapiro-Wilk test to assess normality and a Brown-Forsythe test to determine equal variances. When these two assumptions were met, we performed a 1-way ANOVA test. When one or both of these assumptions were not met, we conducted a Kruskal–Wallis 1-way ANOVA on the ranks test. For our post-hoc analyses, we either performed a parametric Tukey’s all pairwise multiple comparisons procedures, or a non-parametric Dunn’s all pairwise multiple comparisons procedure. The level of statistical significance was set at *p* ≤ 0.05.

## 3. Results

### 3.1. Toxicity Analysis of 3T3-L1 Cells Exposed to MGF and MLT

**Figure 2:**
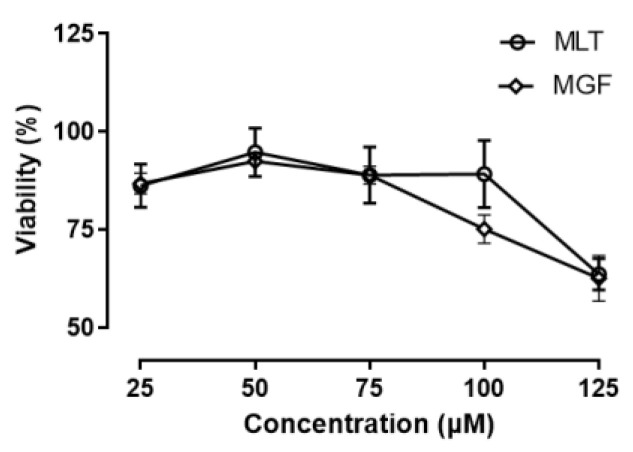
Assessing toxicity in 3T3-L1 cells exposed to mango leaf tea (MLT) and mangiferin (MGF). Cell viability was analyzed using an MTT assay in 3T3-L1 cells treated with either MLT or mangiferin MGF at increased doses for 48 h. Six independent experiments were conducted with triplicate biological replicates analyzed for each experiment. Data are mean ± SEM.

For MGF and/or MLT to be used therapeutically, they will need to be devoid of harmful effects. Thus, to assess the safety profile of MGT/MLT, we analyzed cell viability in 3T3-L1 preadipocytes exposed to these two bioactive compounds. Preadipocyte 3T3-L1 cells were cultured in the presence of MGF (0, 25, 50, 75, 100, and 125 µM) and MLT (0, 25, 50, 75, 100, and 125 µM) for 24 h. When cells were incubated with increasing doses of both MLT and MGF, cell viability remained >75% in cells treated with concentrations of up to 100 µM. However, at a concentration of 125 µM, viability was shown to decrease <75% (Figure 2). Based on these results, we selected a 100 µM dosage of MGF/MLT to treat 3T3-L1 cells to maximize efficacy while preventing excessive cellular toxicity. 

### 3.2. MLT-Mediated Suppression of Intracellular Triglycerides within Cultured Adipocytes

**Figure 3:**
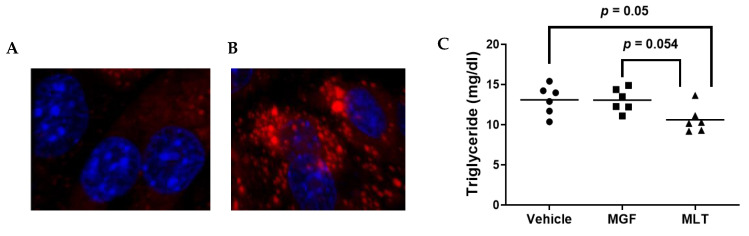
Adipocytes incubated with MLT result in reduced intracellular TG content. (**A**,**B**) Oil-red cell staining and cell nuclei DAPI counterstain of undifferentiated 3T3-L1 cells (**A**) and differentiated 3T3-L1 adipocytes (**B**). (**C**) Intracellular TG levels were quantified using a colorimetric assay to measure intracellular TG content in cultured adipocytes treated with vehicle only, MGF, or MLT. Three independent experiments were performed with duplicate biological replicates assessed for each experiment. Bars are group means.

Triglyceride (TG) is the most relevant glyceride stored in the adipocyte lipid droplet. One sign of adipogenesis is the buildup of lipids in adipocytes [29]. TG is a critical metabolic component of lipid droplets, contributing to obesity. Thus, we performed our experiments using 3T3-L1 in their adipocyte form by adapting a well-established protocol to induce adipocyte differentiation in this cell type. Unlike undifferentiated 3T3-L1 cells, the differentiated cells exhibit large lipid droplets (Figure 3A,B). When we assessed the change in the contents of intracellular TG in the treated adipocytes exposed to either vehicle or MGF/MLT, there were differences observed in TG content among groups, as exposure of 3T3-L1 adipocytes to MLT resulted in a significant decrease in the level of the TG in comparison with the vehicle. Likewise, a trend was observed between MGF and MLT (*p* = 0.054). However, no significant changes between the vehicle and MGF were observed (Figure 3C). Therefore, these results suggest that MLT exhibits TG-suppression effects independent of MGF present in this compound.

### 3.3. MLT Treatment Increases Secretory Adiponectin and Improves Glucose Uptake in Cultured Adipocytes

**Figure 4:**
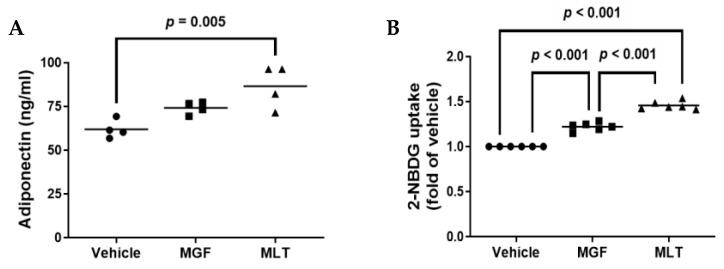
Incubating cultured adipocytes with MLT augments the secretion of adiponectin and enhances glucose uptake. (**A**,**B**) Adipocytes were treated with vehicle, MGF, or MLT. ELISA was used to measure secretory adiponectin (**A**), and a fluorometric kit was used for measuring glucose uptake of fluorescently labeled 2-NBDG (**B**). (**A**) Four independent experiments were conducted using one biological replicate for each experiment. (**B**) Two independent experiments were performed, each with triplicate biological replicates used. (**A**,**B**) Bars are group means.

Body mass index, blood pressure, fasting glycemia, insulin resistance, and serum insulin levels are all inverse relationships with serum adiponectin levels [30]. Adiponectin is one of the most abundantly released proteins from human adipose tissue [31]. In agreement with a previous study [32], we detected a significant difference in the amount of adiponectin secreted in 3T3-L1 cells exposed to MLT or when compared to vehicle control cells (Figure 4A). However, there were no reported significant differences in MGF-treated cells compared to vehicle-treated 3T3-L1 cells. This implies that MLT may demonstrate properties that enhance adiponectin secretion that extends beyond MGF-dependent effects.

For glucose uptake levels in the 3T3-L1 adipocytes, there was a significant increase in glucose uptake among all three groups, as both MGF and MLT treatments resulted in a significant increase in glucose uptake within cultured adipocytes when compared to control cells, and when comparing MGF and MLT treatments, the MLT treated cells demonstrated a significant increase in glucose uptake when compared to the MGF treatment group (Figure 4B). Therefore, these results indicate that MGF directly affects glucose uptake in adipocytes. In addition, other compounds within MLT demonstrate the ability to improve adipocyte glucose uptake even further than MGF alone.

### 3.4. Exposing Cultured Adipocytes to MLT Alters Metabolic Gene Expression

**Figure 5:**
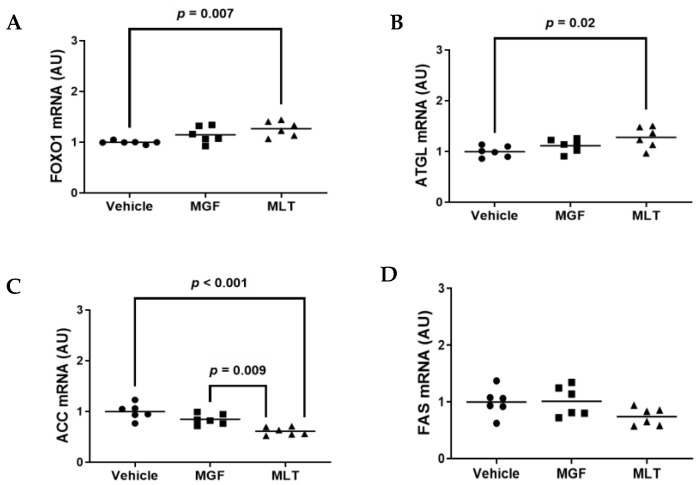
Treatment of cultured adipocytes with MLT modulates the expression of critical genes involved in nutrient metabolism. (**A**–**D**) Adipocytes were exposed to vehicle only, MGF, or MLT, and mRNA expression of FOXO1 (**A**), ATGL (**B**), ACC (**C**), and FAS (**D**) was analyzed. Three independent experiments were conducted using duplicate biological replicates for both experiments. AU = arbitrary units. Bars are group means.

The process of adipocyte development is regulated by molecular and cellular processes [33,34]. Adipogenesis results in changes to the expression of several transcriptional factors, adipogenesis-specific markers, and genes involved in de novo lipogenesis and gluconeogenesis [33,35,36,37,38]. Thus, we investigated whether MGF and/or MGF regulates adipogenic genetic expression levels of FAS, FOXO1, ATGL, and ACC via RT-qPCR (Figure 5A–D) by using the primers pairs listed within Table 1.

FOXO1 inhibits adipocyte differentiation via insulin signaling [39]. ATGL is one of the main rate-limiting enzymes in triglyceride lipolysis, which account for a predominant proportion of the triglyceride hydrolysis activity of adipose tissue [40]. ACC and FAS are vital enzymes for the de novo lipogenesis pathway [14,41]. We identified significant changes in the mRNA expression levels of FOXO1, ATGL, and ACC in cells incubated with MLT compared to vehicle control cells (Figure 5A–C). A significant increase was observed between MLT-treated and vehicle-treated 3T3-L1 cells for FOXO1 and ATGL (Figure 5A,B). In contrast, ACC was significantly decreased in MLT- and MGF-treated cells compared to vehicle-treated 3T3-L1 cells (Figure 5C). However, no significant differences were reported among the three treatments for FAS mRNA expression (Figure 5D). Therefore, these results indicate that MLT may demonstrate the ability to regulate adipogenesis by altering glucose/insulin-responsive genes in adipocytes.

## 4. Discussion

Inhibition of adipogenesis can be applied as a key for hindering adipose tissue enlargement and can be used as a strategy for obesity management. Therefore, the effect of MLT on the 3T3-L1 preadipocyte differentiation was investigated. As a result, MLT inhibited adipogenesis accompanied by decreased triglyceride accumulation, increased adiponectin, attenuated gene expression of ACC, and increased FOXO1 and ATGL gene expression in 3T3-L1 adipocytes.

ACC and FAS are essential enzymes for synthesising endogenous fatty acids, which are potentially crucial in fat deposition [14]. Likewise, ATGL, along with hormone-sensitive lipase (HSL), are two main rate-limiting enzymes in triglyceride lipolysis [42,43,44], which primarily account for triglyceride hydrolysis activity within adipose tissue [40]. In vitro, studies have demonstrated that the rate of lipolysis in adipocytes is associated with ATGL expression levels [45,46]. In this regard, SIRT1 could regulate lipolysis in adipocytes by FOXO1-mediated expression of ATGL [47] and thus may accelerate fatty acid lipolysis. Since SIRT1 appears crucial for regulating fatty acid metabolism in adipocytes, future studies should investigate whether MLT impacts SIRT1 activity in adipose tissue.

In our in vitro study, MLT reduced TG content and increased secretory adiponectin in 3T3-L1 adipocytes while downregulating ACC but upregulating both FOXO1 and ATGL mRNA expression. These findings parallel the results of prior studies. For instance, a previous report examined the effects of mango leaf extract on adipocytes. These experimental findings show that mango leaf extract increases adiponectin levels while reducing intracellular lipid content. Furthermore, mango leaf extract decreased the expression of genes associated with lipid metabolism, including FAS, PPARG, DGAT1, DGAT2, and SCD-1 [32]. Another study showed phenolic compounds isolated from mango leaves upregulate adipocyte AMPK and downregulate the expression of FAS, HSL, and SREBP-1c [48], which are all crucial for TG biosynthesis [48]. A previous publication also reported that MLT extracts exposure to adipocytes downregulated the expression of ACC, HSL, and FAS [42]. Those findings provide promising evidence for the potential effectiveness of mango leaf in correcting deleterious lipid homeostasis. We believe that the additional data in our study further augment these prior results since we are the first to report that MLT extracted from brewed mango leaves exhibits anti-obesity properties precisely in adipocytes. Indeed, we deem this finding both innovative and novel, as tea steeping is an internationally used method for tea preparation. Hence, consuming brewed tea from mango leaves may be an easy, inexpensive, and convenient method to help combat obesity.

From our results, we were surprised about the MGF treatment as a standalone not having stronger positive biological effects within the treated cultured adipocytes when compared to the cultured adipocytes treated with MLT. One possibility, for this reason, is that other biological compounds within the MLT exhibit stronger positive anti-audiogenic effects when compared to MGF. When considering the several other bioactive components present in MLT [15], some of these bioactive compounds may indeed have more potent anti-adipogenic effects than MGF. Another possibility about MLT appearing to have superior anti-adipogenic activity when compared to MGF alone is that there may be additive or synergistic anti-adipogenic properties of the ingredients within MLT when delivered with MGF simultaneously, which may explain why we observed a more substantial anti-adipogenic effect in cultured adipocytes exposed to MLT when compared to adipocytes treated with MGF as a standalone. When considering this, future studies should be devoted to meticulously identifying which bioactive compounds in MLT demonstrate the most potent anti-adipogenic effect and whether these constituents display synergy when precisely delivered to adipocytes simultaneously.

The main limitation of our study is that we failed to investigate any mechanistic underpinnings related to the biological effects in 3T3-L1 adipocytes exposed to MLT/MGF. Additionally, results from 3T3-L1 adipocytes may not be easily translatable in vivo. From our results, we also cannot distinguish the effects observed from MLT, which are independent of MGF, as we did not include a treatment group which consisted of 3T3-LI adipocytes treated with MLT devoid of MGF. Thus, future studies should be considered, which involve testing whether MGF, MLT, and MLT minus MGF impact lipid metabolism in animal models.

## 5. Conclusions

In conclusion, our findings suggest MLT accelerates lipolysis and suppresses lipogenesis via decreasing triglyceride accumulation, increasing adiponectin, increasing glucose uptake, downregulating ACC, and upregulating FOXO1 and ATGL in 3T3-L1 adipocytes. Therefore, these results imply that ingestion of MLT may attenuate adipose tissue accumulation. Indeed, inhibition of adipogenesis from MLT may hinder adipose tissue enlargement and potentially be used to manage obesity clinically.

## Figures and Tables

**Table 1:** qRT-PCR primer pairs used to measure mRNA expression.

Target	Sequence (5′-3′)	Amplicon (bp)	Accession Number
GAPDH	Forward: AACTTTGGCATTGTGGAAGG	132	NM_001289726
	Reverse: GGATGCAGGGATGATGTTCT		
FOXO1	Forward: GATCTACGAGTGGATGGT	523	NM_019739
	Reverse: CAGCGTAGACGCCATCTT		
ATGL	Forward: CACTTTAGCTCCAAGGATGA	380	NM_001163689
	Reverse: TGGTTCAGTAGGCCATTCCT		
ACC	Forward: GGGCTACCTCTAATGGTCTT	439	NM_133360
	Reverse: CTACCTGATGGTAAATGGGA		
FAS	Forward: TTGCTGGCACTACAGAATGC	192	NM_007988
	Reverse: AACAGCCTCAGAGCGACAAT		

## Data Availability

All data in the study is provided within the manuscript.
